# Adherence to isoniazid preventive therapy among child contacts in Rwanda: A mixed-methods study

**DOI:** 10.1371/journal.pone.0211934

**Published:** 2019-02-11

**Authors:** Francine Mwayuma Birungi, Stephen Michael Graham, Jeannine Uwimana, Angèle Musabimana, Brian van Wyk

**Affiliations:** 1 Department of Epidemiology and Biostatistics, School of Public Health of the College of Medicine and Health Sciences, University of Rwanda, Kigali, Rwanda; 2 Faculty of Community and Health Sciences, University of the Western Cape, Bellville, South Africa; 3 Centre for International Child Health, University of Melbourne Department of Paediatrics and Murdoch Children's Research Institute, Royal Children's Hospital, Melbourne, Australia; 4 International Union Against Tuberculosis and Lung Disease, Paris, France; 5 Centre for Evidence-Based Health Care, Division of Epidemiology and Biostatistics, Department of Global Health, Faculty of Medicine and Health Sciences, Stellenbosch University, Tygerberg, South Africa; 6 School of Public Health of the College of Medicine and Health Sciences, University of Rwanda, Kigali, Rwanda; Médecins Sans Frontières (MSF), SOUTH AFRICA

## Abstract

**Background:**

The World Health Organization recommends isoniazid preventive therapy (IPT) for six months for child contacts without tuberculosis (TB), who are exposed to an adult with active TB. The effectiveness of IPT depends on 80% or greater adherence to medication. In the current study, we assessed IPT adherence and explored barriers to and facilitators of adherence among eligible child contacts in Kigali, Rwanda.

**Methods:**

A mixed method study design was used to prospectively assess adherence to IPT among eligible child contacts and its associated factors through a quantitative, observational cohort study, and to explore barriers to and facilitators of adherence to IPT through a descriptive qualitative study.

**Results:**

Of the 84 child contacts who started IPT, 74 (88%) had complete adherence and ten (12%) had incomplete adherence. There were no factors (individual characteristics of index cases, households and or health facility characteristics) found to be significantly associated with IPT adherence in the bivariate and multivariate analysis. In the qualitative analysis, we identified factors relating to parents/caregivers, disease, household and health-care providers as major themes determining IPT adherence.

**Conclusion:**

There was a high rate of IPT completion in this cohort of eligible child contacts living in Kigali. However, structural factors (poverty and relocation) were found to be the main barriers to IPT adherence that could be addressed by health-care providers.

## Introduction

Young children exposed to an adult or older child with tuberculosis (TB), referred to as an index case [1], are at high risk of infection with *Mycobacterium tuberculosis* [2,3]. Without any intervention, 5–10% of infected children will develop active TB within one year, and the risk is the highest among the youngest (<2 years old) or HIV-infected children [2,4]. Also, infants and young children are at high risk of developing severe disseminated forms of TB such as TB meningitis and miliary TB, and of TB-related mortality [5,6].

The World Health Organization (WHO) has for many years recommended isoniazid preventive therapy (IPT) for at least six months for young (<5 years) children who are exposed to a TB index case and who do not have active TB disease [7,8]. More recently, TB preventive treatment has received greater attention as a key element of the WHO's End TB strategy, which aims to reduce TB incidence by 90% by 2035 [9]. The most recent WHO guidelines for treatment of latent TB infection now include the consideration of older (≥5 years) child contacts without active TB for preventive therapy [10]. IPT has been proven effective in reducing TB incidence [8,9] and is included in the national TB programme (NTP) guidelines of many resource-limited countries [10]. However, IPT is not consistently offered to at-risk children, and when offered, is often unsupervised and characterised by poor uptake and adherence [11–14]. The effectiveness of IPT is dependent on 80% or greater adherence to medication [8]. Several studies from Indonesia, Ethiopia, Brazil and South Africa show inadequate adherence to IPT among child contacts [14–18]. Among these, the two studies conducted in Indonesia [16,17] revealed that access, social support and regime, caregivers and health care related factors were barriers and facilitators to IPT adherence. Studies from Indonesia and Brazil also reported that transport and medication costs were associated with incomplete adherence [16,18].

Rwanda established the IPT policy in 2005, and recently the NTP has focussed on TB in children because case detection and TB treatment are recognised as an opportunity to reduce child mortality [19,20]. Since 2009, the NTP strategy has been promoting the uptake and adherence to IPT as one of the 30 performance indicators. However, to date, no study has been conducted in Rwanda to assess the IPT adherence among child contacts, and little is known about the factors associated with inadequate adherence.

In Rwanda community health workers (CHWs) are involved in the management of child contacts. Other local interventions include free TB care, increased number of primary health-care centres (PHCs) and a community-based health insurance scheme to increase geographic and financial access to health care. In addition, performance-based financing has been implemented to motivate health-care providers to improve service output and quality of care. The current study reports on IPT adherence and explore s the facilitators and barriers to IPT adherence in Kigali, Rwanda.

## Methods

### Settings and participants

This study is part of a cross-sectional research project that was conducted in Kigali, the capital of Rwanda by a consortium of researchers from South Africa, Australia and Rwanda between 1 August 2015 and 29 February 2016 [21]. The study aimed to evaluate the diagnostic performance of the Xpert MTB/RIF assay in sputum collected by the gastric Lavage (GL) technique from symptomatic child contacts. Kigali has 35 primary health care centres (PHCs) that offer tuberculosis (TB) diagnosis and treatment services, which are also regarded as entry points for TB care. Thirteen of these PHCs were included in the main study based on them meeting the criterion of recording at least ten sputum smear-positive pulmonary TB (PTB) between January and June 2015.

Overall, 346 index cases of sputum smear-positive PTB were diagnosed and treated for the main project. Of these, 136 (39%) had at least one child contact who was younger than 15 years old at the time of the study. The 136 index cases had 270 child contacts. From the 136 index cases, 105 (77%) met the inclusion criteria of index cases and had 216 child contacts who met the inclusion criteria for the main project.

Of the 105 index cases, only those whose child contacts started IPT were recruited for the present prospective study that was conducted between August 2015 and August 2016. Eligible child contacts for the current study were younger than 5 years old, who started IPT according to the WHO recommendations [22]. They also shared the same households with the selected index cases in the three months before diagnosis of the latter. Only children who had their parental or primary caregivers’ written consent were enrolled in the present study. Child contacts born after the index cases were diagnosed and had initiated TB treatment, child contacts on TB treatment, and those who were not living in the same households as the index cases before diagnosis were excluded from this study.

This study presents specific data elements that were derived from the main project. Those elements can influence IPT adherence which is under study in the current research. They include the characteristics of index cases, child contacts, households, health facility, TB screening results together with physical and chest X-ray (CXR) results [21].

### Study design

In this study, we used a mixed research method design to prospectively assess IPT adherence and outcomes among child contacts through a quantitative, observational cohort study. Furthermore, we explored barriers to and facilitators of adherence to IPT through a descriptive qualitative study [23].

### Data collection for the quantitative component

In August 2015, each TB focal person based at the selected PHC used a specific form, provided by researchers, to record each time the child came to collect a month’s supply of IPT. A TB focal person, usually an experienced nurse working at the PHC, is responsible for coordinating and managing all activities such as providing TB treatment or IPT, contact screening, reporting, follow-up and supervision of TB patients and contacts.

Before data collection, TB focal persons from the selected PHC were trained on data collection procedures for two days. The training aimed at equipping them with skills to provide follow-up care (for example, monthly screening and transfer to the next level when a child has symptoms suggestive of TB) of children on IPT [S1 Appendix].

The researchers requested the parents/caregivers of child contacts who had been initiated on IPT to visit the PHC for clinical evaluation and receive the next month’s supply of IPT each month until the end of the treatment. In this study, researchers measured “adherence” through a monthly collection of isoniazid [16]. “Complete adherence” refers to the collection of six of the child’s monthly prescriptions, whereas “incomplete adherence" means that the child had received/collected less than six of his/her monthly prescriptions. IPT failure in this study is defined as a proportion of child contacts on IPT who developed TB during the monitoring period. To achieve this, the researchers monitored all eligible child contacts for symptoms suggestive of TB such as persistent one-week fever (>1 week), cough (>2 weeks), weight loss, night sweats for 12 months following the initial evaluation.

During the six months of monitoring, while receiving IPT, the TB focal person screened the child contacts for TB at each visit for the presence of symptoms suggestive of TB, using the IPT form [S1 Appendix]. For the second six months’ follow-up (post-IPT), the TB focal person evaluated the child contacts at 3 months and 6 months after finishing IPT. The researchers provided transport fees to all parents/caregivers who brought child contacts for screening during those two visits, which were not part of the routine clinical follow-up and monitoring. The post-IPT (at six months) visits were done to evaluate the impact of the IPT.

During the follow-up, the TB focal person referred any child contact showing symptoms suggestive of TB to the district hospital for further TB evaluation including smear microscopy, Xpert MTB/RIF assay and solid culture using sputum collected through gastric lavage. If a child contact was diagnosed TB positive, he/she was treated according to the national guidelines.

### Data collection for the qualitative component

The researchers carried out in-depth interviews with 23 parents/caregivers of child contacts and ten health-care providers working in the TB service. Three focus group discussions (FGDs) were held with 24 CHWs who provided TB support in the community.

The researchers used purposive sampling to select parents/caregivers from the different catchment areas around the participating PHC to represent child contacts with complete adherence. They also used purposive sampling to select TB focal persons to represent different districts and types of PHCs (faith-based and public PHCs). The study included all available parents/caregivers with incomplete adherence.

Telephone numbers of parents/caregivers of child contacts available from the previous study database [21] and those of CHWs provided by TB focal persons, were used to inform and invite participants about their selection in this study. If in agreement, they were requested to go to the nearest PHC at a time and date indicated by the researchers.

Fieldworkers conducted interviews with parents/caregivers whose children had complete adherence and the health facilities staff until the data saturation was achieved (i.e. until no new data emerged). However, existing themes could accommodate new findings [24]. Each FGD involved eight CHWs who were purposefully selected to represent the different districts and PHCs in line with Krueger methodology [25].

Three fieldworkers (female senior nurses) experienced in qualitative methodology were recruited to conduct interviews in the local language using interview guides designed for this specific study. Two days of debriefing sessions were held with the qualitative fieldworkers before the fieldwork started. In the debriefing sessions, the principal investigator discussed each question with them explaining the nature of the response that each one was meant to elicit. Discussions on how to probe for further explanations were also held.

Different interview guides were used for parents/caregivers and health-care providers (TB focal persons and CHWs). Parents/caregivers whose child contacts had incomplete adherence were interviewed to explore possible barriers related to IPT access. The questions were framed to avoid apportioning blame for non-collection of IPT for their children as recommended by the Ethics Review Board of the University of Rwanda, College of Medicine and Health Sciences.

Interview guides were pretested, and no further modification was needed. The pre-test was done using four parents/caregivers as participants (two whose child contacts had IPT complete adherence and two others whose child contacts had incomplete IPT adherence) and one TB focal person from a non-selected PHC based in Kigali. Parents/caregivers involved in the pre-test were identified by the TB focal person where the pre-test was done.

Participants signed a written informed consent form after reading it and after the researchers had read an introduction explaining the purpose and benefit of the study. The interview with health facility staff took place at their workplace (in the absence of their superior or any other staff), and the one with parents/caregivers took place at the PHC nearby their homes. These interviews lasted between 45 minutes to one hour. None of the participants declined to participate in the interview.

Depending on the participants (parents/caregivers, TB focal persons or CHWs) answering the questions, the researchers investigated their experiences about providing or receiving IPT. They also investigated barriers to and facilitators of IPT adherence, and expectations and suggestions. Two experienced qualitative researchers conducted the FGD in the local language at the PHC proposed by the selected CHWs. These fieldworkers used a discussion guide during the FGD; which lasted between 1½ to 2 hours.

After obtaining their agreement, the fieldworkers, under the supervision of the principal investigator, audio-recorded all interviews and FGD. For quality control, at the end of each interview session, the fieldworkers summarised the salient points of the interviews with confirmation or adjustments from the participants when necessary. Hereafter, the fieldworkers fully transcribed interviews and FGDs in Kinyarwanda. Afterwards, the principal investigator checked the transcriptions and carried out necessary alterations before analysis. The transcripts were then translated into English by a qualified translator. The English transcripts were verified by a bilingual member of the research team to ensure that these were clear, and participants' views adequately reported.

### Data analysis and management of the quantitative component

The researchers double-entered the quantitative data into a Microsoft Excel worksheet and exported these to STATA13 Software [26] for statistical analysis after checking their consistency. Continuous variables, such as the age of the child contacts or monthly household income, were categorised following epidemiological or economic constructs. Age of child contacts was dichotomised into two values (≤2 years and >2 years) as the literature suggests that infants who are ≤2 years old are more likely than those >2 years old to acquire TB [2,4]. The variable monthly household income was categorised in two values (Incomes ≤50.000 and 50.000 Rwandan Franc) following the poverty headcount ratio of Rwanda in 2018, which is $1.90 a day [27], equivalent to 50, 000 Rwandan Francs.

Researchers then analysed the data using descriptive and multivariate statistics, described categorical variables using frequency tables and proportionate methods. The researchers further performed the univariate and multivariate logistic regressions to determine characteristics associated with IPT adherence. Where appropriate, the researchers performed the Chi-square test or Fisher's exact test to assess the association between two variables and included those variables with a p-value <0.2 in univariate analysis in the logistic regression model using backwards stepwise method.

The final model included the following variables: sex of the child, child contact' s HIV status, sleeping in the same room as index case, the index case’s sex and HIV status, the income of the household, if the head of the household had knowledge of IPT protection and attitude of health providers towards patients. This further included calculating the odds ratios (OR) and adjusted OR along with its 95% confidence interval using STATA13 software [26]. In this case, the researchers declared the associations as significant if the p-value was <0.05.

### Data analysis and management of the qualitative component

Two researchers analysed the qualitative data using thematic analysis as described by Braun et al. [28]. They repeatedly read the transcripts for full immersion and carried out an inductive thematic analysis using Atlas.ti-7 software [29]. Two researchers coded portions of the transcripts together. Discrepancies were discussed and resolved by consensus. Then they grouped codes into sub-themes and organised them under themes.

### Ethical approval

The Biomedical Research Ethics Committee of the University of the Western Cape and the Ethics Review Board of the University of Rwanda, College of Medicine and Health Sciences approved the study protocol. Permission was obtained from Rwanda NTP to collect data from the eligible PHC. The researchers assured the participants’ anonymity and confidentiality: for the focus group discussions. Numbers were allocated to each participant at the start of the discussions, and they were asked to refer to one another according to these. Regarding the in-depth interviews, during transcription, pseudonyms were used to ensure the identity of the participants remains anonymous. All records were stored in a password-protected folder in the computer, and the hard copies of the data (printed transcripts) were locked at the School of Public Health of the University of Rwanda, College of Medicine and Health Sciences (SPH-CMHS-UR in a cupboard accessible only to the principal investigator who is the employee of the university of Rwanda.

## Results

### Quantitative results

**Fig 1:**
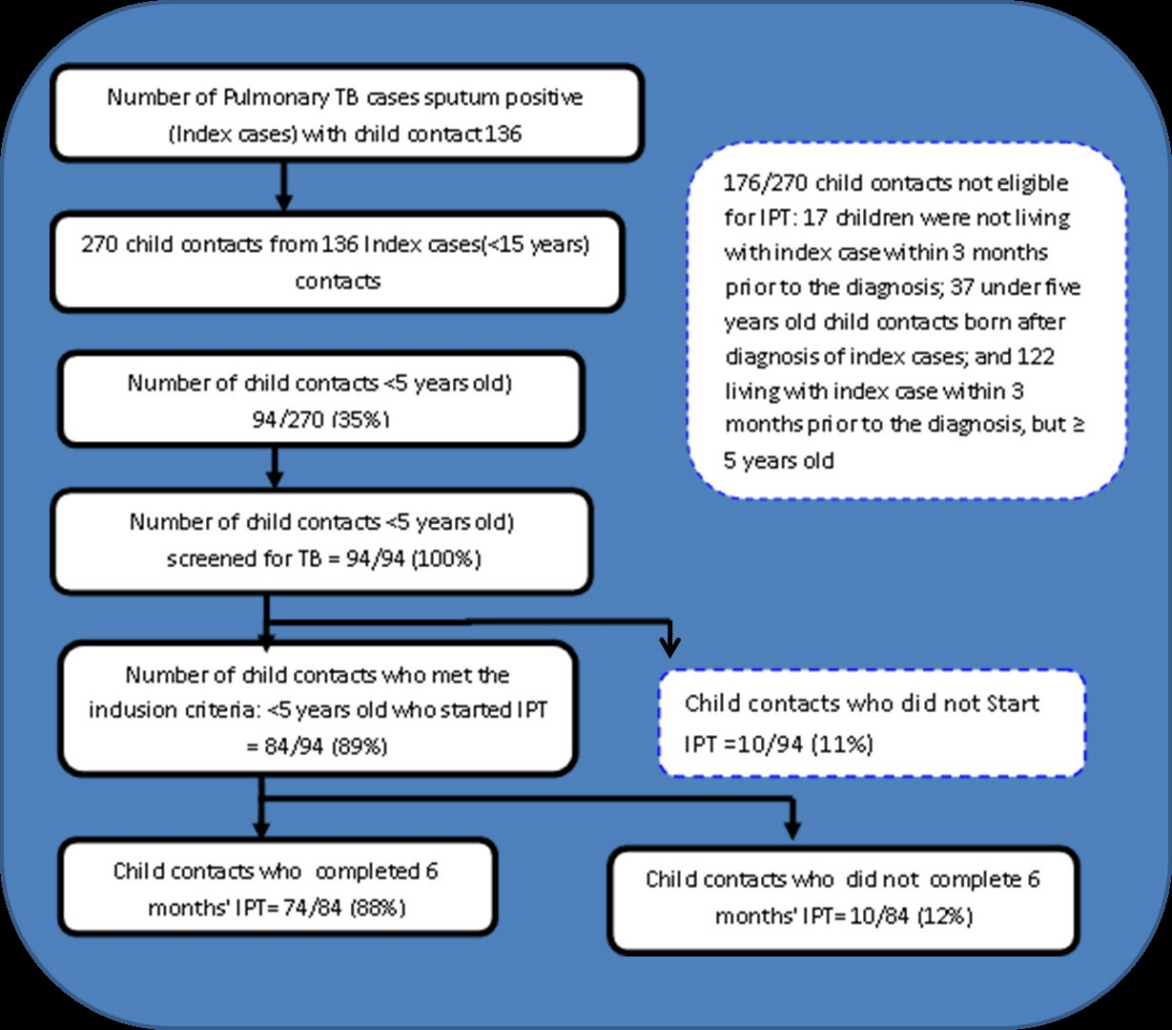
Flow diagram of child contacts from recruitment to IPT completion.

**Fig 2:**
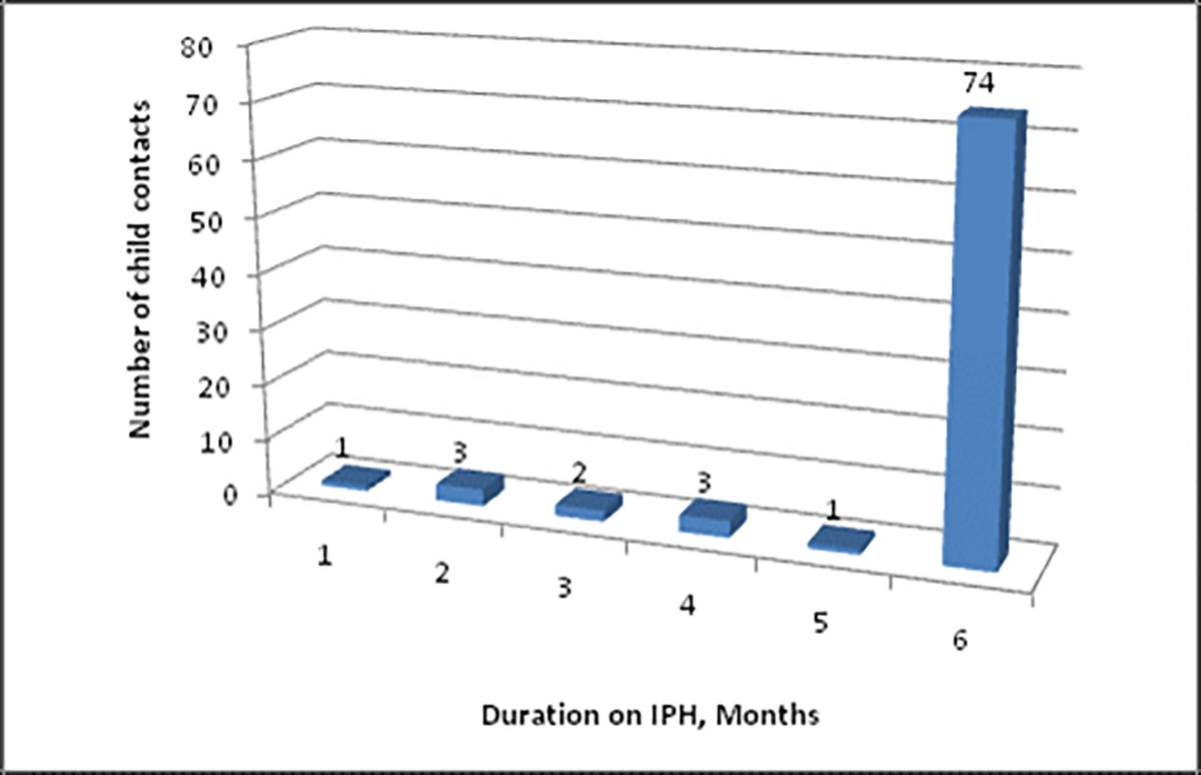
Distribution of number of months IPT prescription was collected.

Among 270 below 15 year-old child contacts recruited from 136 eligible sputum smear-positive PTB index cases (n = 346) diagnosed and treated at 13 PHCs in Kigali, 94 (35%) child contacts from 72 index cases were below five years old and eligible for IPT. To evaluate adherence in this study, 84 (89%) who started the IPT were enrolled from 61 index cases. As shown in Fig 1, 74 (88%) completed six months of IPT, with only ten (12%) who did not complete the treatment. Fig 2 shows the number of months for which child contacts who were initiated into IPT failed to complete the 6 months treatment.

The characteristics of child contacts who started IPT are shown in Table 1. There was no statistically significant difference in the characteristics of children who completed six months of IPT compared to those who did not.

**Table 1:** Characteristics of child contacts who started isoniazid preventive therapy by adherence group (N = 84).

Characteristics	Total (%)	Complete Adherence (n = 74)	Incomplete Adherence (n = 10)	*P-value*
** Age group** ≤ 2 Years	37/84 (44)	32/74 (43)	5/10 (50)	0.69
**Sex** ** **Female	37/84 (44)	30/74(41)	7/10 (70)	0.07
** BCG scar present** Yes	80/84(95)	70/74(95)	10/10(100)	0.59
** Tested for HIV** Yes	39/84 (46)	32/74 (43)	7/10 (70)	0.17
** HIV test Result** Positive	2/39 (5)	1/32 (3)	1/7 (14)	0.10
** Relationship to Index case**				0.10
** **Child	65/84 (77)	55/74 (74)	10/10 (100)	
** **Others	19/84(23)	19/74 (26)	0	
**Had symptoms suggestive of TB during the initial screening**	19/84 (23)	17/74 (23)	2/10 (20)	0.83
** Share the same bedroom with index cases** Yes	42/84 (50)	36/74 (49)	6/10 (60)	0.73

BCG = bacille calmette guerin; IPT = isoniazid preventive therapy, TB = tuberculosis; HIV = human immunodeficiency virus.

In Table 2, the characteristics of child contacts, index cases, households and health facilities are listed. None of the characteristics we evaluated was significantly associated with incomplete adherence to IPT in the bivariate and multivariate analysis.

**Table 2:** Risk factors for non-adherence to isoniazid preventive therapy.

Factors	TOTAL (%)	OR (95%; CI)
**Child contacts (n = 84)**		
Female	37 (44)	3.4 (0.14–3.7)
HIV positive	2/39 (5)	5.1 (0.28–94)
Not sleeping in the same room as the index case	35 (42)	0.3 (0.06–1.5)
**Index cases (n = 61)**		
Female	28 (46)	3.4 (0.81–14.3)
HIV-positive	13/51 (25)	0.3 (0.05–1.36)
**Household factors (n = 61)**		
Income >50.000 Rwandan Franc	23 (38)	0.3 (0.08–1.95)
No knowledge of IPT protection^a^	38 (62)	5.5 (0.65–45)
**Heath facility factors (n = 61)**		
Provider not friendly	1/61 (1.6)	8.1 (0.46–141)

IPT = isoniazid preventive therapy; HIV = human immunodeficiency virus; CI = confidence interval; OR = odds ratio

^a^ Not knowledgeable about the administration of INH for six months to protect child contacts against TB.

Only one (1.2%) of the 84 child contacts who started IPT developed TB six months after completing the full 6-month IPT, i.e. at 12 months following initial screening and uptake. He was a 3-year-old male, HIV uninfected, who had a clinical diagnosis of TB based on history, physical examination, and CXR. He had TB-related symptoms at the time of initial screening, but further clinical evaluation and CXR were negative for a diagnosis of TB. He remained asymptomatic while on IPT.

### Qualitative findings

Interviews were conducted with ten TB focal persons from selected PHCs and 15 parents/caregivers whose children had complete adherence, and eight whose children had incomplete adherence. The characteristics of parents/caregivers are presented in Table 3.

The FGDs were attended by 24 CHWs, which included eight participants from each district.

**Table 3:** Demographic characteristics of parents/caregivers.

Characteristics	Complete adherence (n = 15)	Incomplete adherence (n = 10)
**Age in years, median (IQR)**	36 (22–63)	33 (28–43)
**Relationship to child**		
** **Mother	11	7
** **Father	2	0
** **Grandmother	2	1
**Education level**		
** **Never attended school	5	0
** **Primary school	5	6
** **Secondary school	4	2
**Socio-economic status**		
** **Low	8	6
** **Middle	7	2
**District of residence**		
** **Nyarugenge	5	1
** **Kicukiro	2	2
** **Gasabo	8	5
**Relation to Index case**		
** **Herself	6	2
** **Wife	6	6
** **Others	3	0

IQR **=** interquartile range

**Fig 3:**
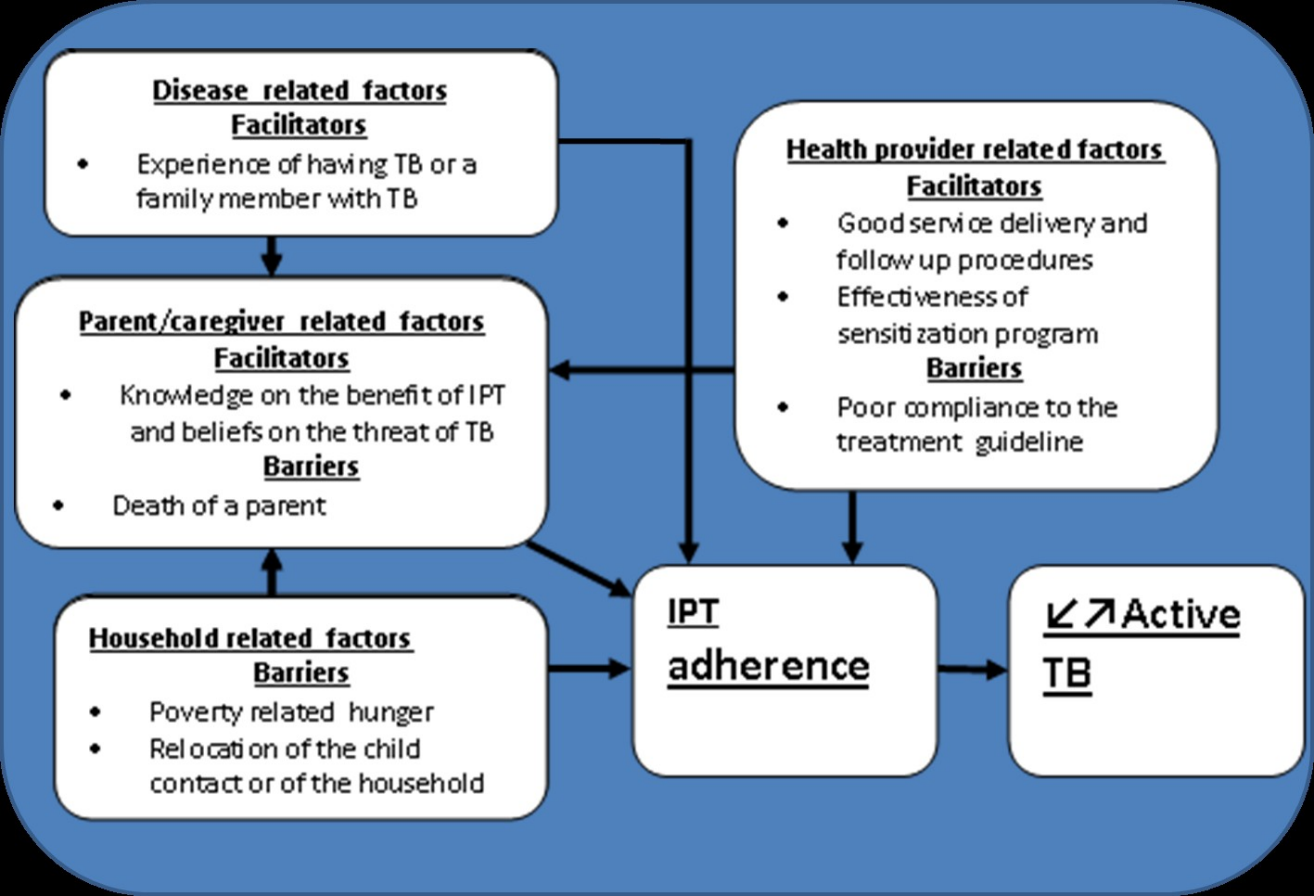
A framework mapping factors influencing isoniazid preventive therapy adherence in Kigali, Rwanda.

The barriers to and facilitators of the IPT adherence, with themes and sub-themes, are presented in Fig 3. The figure has four boxes and each box represents a theme. Also, each bullet in a box represents a sub-theme which can be a facilitator or barrier to IPT adherence.

The reported facilitators of IPT adherence included themes around parents/caregivers, disease, and health-care provider-related factors which are described in detail below.

#### Parent-/caregiver-related factors

Parents/caregivers’ knowledge about the benefit of IPT and beliefs about the threat of TB disease were reported as a facilitator of IPT adherence.

*I knew that this medicine protect*s *my child from getting TB*. *I know that TB is a bad disease*, *so I put that programme (to give him IPT) among my obligations*. (Mother, 29 years, complete adherence)

#### Disease-related factors

Overall parents/caregivers whose children had complete adherence mentioned their own TB disease experience or experience taking care of a relative with TB as the core factor motivating them to provide IPT to their children as recommended by health-care providers.

*I'm telling you*, *from my experience when you have experienced TB disease*, *you cannot really wish to see your child contaminated and feel what you have experienced*, *and hence that fear helps you to give the medicine as prescribed by the doctor to protect the child from contamination*. (Mother, 33 years, complete adherence)

*I saw how seriously sick my mother-in-law was when she was struggling with the TB*, *which had evolved into multi-drug resistant TB; and I was the one who took care of her*. *Recalling that situation pushed me to act quickly and get medicine for my children*. *I always made sure they took it as prescribed*. (Mother, 40 years, complete adherence)

#### Health-care providers’ factors

Most of the participants reported on the positive support by health-care providers and CHWs as facilitators of IPT adherence. They commended health-care providers for the way they taught, provided them with information and education on the IPT adherence and for their successful follow up.

*It is the nurses in charge of the follow-up of the TB patients who often go on the fields*, *and we (CHWs) make them visit people to whom we give IPT and TB treatment*. *They also inquire about their [TB patients*, *child contacts] health status*, *ask them questions about how they take their treatment or give children treatment*. *This [follow up] could have increased the number of child contacts who finished IPT*. (CHW6, Nyarugenge district)

*It's CHWs who help those (child contacts) to take their medicine and also us (TB focal person)*. *We supervise CHWs many times because if we gave them medicine*, *they (CHWs) are supposed to make sure that children take it well and on time*. *When we visit them (CHWs)*, *we check if they gave the medicine to children as we prescribed*. (TB focal points 1, Public PHC)

*Through sensitisation programmes conducted by nurses*, *we are well informed that immunisation protects our children from contracting a disease*. (Mother, 31 years, complete adherence)

We also explored the barriers to IPT adherence. The reported barriers included themes around caregivers, household and healthcare factors which are described in detail below.

#### Factors related to parents/caregivers

Parents/caregivers whose child contacts had complete adherence and some health-care providers reported that the death of a parent/caregiver led to the child being placed in another family, whose members did not share the importance of continuing the adherence to IPT or were living too far from the PHC.

…*sometimes a mother who was following her child’s treatment well can die before the treatment is complete*, *and those who take care of the child may neglect to continue the child's treatment*. (Health-care provider, 35 years, public PHC)

*There are children who can have the tragedy of losing their parents when on IPT*. *For example*, *in my village*, *there was a woman whom I was giving TB treatment*, *and she had a child who was on IPT*. *That woman died*, *and we buried her in her province*. *This means the child was taken by his mother’s relatives and we did not know who took him to allow us to continue with the follow-up*. *Such kind of child contacts is included among those who did not finish the IPT*. (CHW4, Nyarugenge district)

Parents/caregivers' belief that medications taken without food are harmful was reported as a barrier to IPT adherence by some CHWs who supplied IPT at the community level. Parents/caregivers believe that a hungry child could not be given medicine because it is difficult and harmful.

*It is quite a challenge when you come to give a child his/her medicine you are told by a parent/caregiver*: *please stop* … *stop*! *The child has not eaten anything since the previous day and looks concerned*. *The child has gone hungry and yet has to take medicines and*, *as you know*, *it is not easy to swallow pills/tablets even for an adult and it is more difficult for a child and also harmful to take medicine on empty stomach*! (CHW4, Nyarugenge district)

#### Household-related factors

Many of the parents/caregivers whose child contacts had incomplete adherence reported poverty and relocation as the foremost barriers to IPT adherence. Parents/caregivers reported that poverty led to a lack of food, therefore by necessity, they gave priority to getting a job and being able to provide food for their children rather than going to the PHC to collect medication.

*Sometimes you ask yourself where the meal for the child will come from if I take the child to the PHC*. *Because of this*, *you may decide to look for a job today and plan to take the child to the PHC tomorrow*. *But still*, *you may also fail to get the job that day*, *and that will compel you to try again the following day*. *Finally*, *you will not find any time and stop the treatment altogether*. (Mother, 34 years, incomplete adherence)

The relocation, either of a child contact or the household, was reported as a barrier to IPT adherence. Some participants reported that parents/caregivers are often compelled to place their children with relatives.

*It may happen that you start taking medicine; before its completion*, *you move to another place and find yourself in a situation where you are not able to pay for transport to go to the place where you used to get the medicine from*. *That is what happened to me*! (Mother, 30 years, incomplete adherence)

*For example*, *a mother may start giving her child the medicine*, *but only halfway to completing the treatment*, *she may come and tell you that she does no longer live with the child*, *that she has sent him/her to his/her grandmother’s*. *In that case*, *you understand that the child stops taking the medicine*. (Health-care provider, 30 years, Public PHC)

#### Health-care providers’ factors

The lack of compliance with the treatment guideline by health-care providers was reported as a barrier to complete adherence by a parent/caregiver whose child had incomplete adherence.

*This is something that I myself experienced*. *My child didn’t complete the six months of treatment*, *because when I finished my dose*, *I was told to stop his treatment too*, *although he started it one month later than I did*. *You do understand that the decision to stop the medicine was not mine; it was rather the decision of the nurses*, *who convinced me that my child was no longer running any risk since they followed me up to my full recovery*. (Mother, 28 years, incomplete adherence)

## Discussion

The rate of complete IPT adherence of 88% in this study is higher than the range of adherence rates 26%-76% reported elsewhere [14,16–18,30–32]. To be more precise it is comparable to the 86% and 94.5% rates reported for Benin [33] and the Gambia [34], respectively. IPT adherence is often poor, and a recent systematic review did not identify a particular intervention to improve implementation [35]. However, the successful delivery of IPT may be setting-specific relying on system factors that may be completely different from other similar studies but in different settings such as urban Indonesia [18]. In Rwanda, the government's commitment through NTP to implement local interventions, especially those targeting to improve IPT adherence, such as performance-based financing, free TB services and treatment, increasing the number of PHCs, and involving CHWs in the management of child contacts. The findings from the qualitative study support this assumption. Factors such as financial challenges regarding medication collection including the cost of medication and transport, and long waiting times that were reported as barriers to IPT adherence in other countries where such interventions are not implemented [16–18], were not reported by participants in this study.

Parents/caregivers’ own experience concerning TB disease or their experience of taking care of a relative with TB has been identified as one of the main factors facilitating IPT adherence. The fear to see their offspring suffering from TB, a disabling and killer disease, has been a primary factor motivating them to make sure that their children had complete IPT adherence. This is consistent with a study conducted in Indonesia [17] where the experience of having a family member with TB was found to be a factor in facilitating IPT adherence.

The effective sensitisation programme, service delivery (for example, friendly health providers, supportive and providing all the needed information, especially information on the benefits of IPT or length of treatment) and follow-up procedures have been identified as facilitators of IPT adherence in this study. This finding reinforces the quantitative result that revealed that only one parent/caregiver experienced the health-care providers to be unfriendly. Furthermore, only one parent/caregiver whose child had incomplete adherence reported a lack of compliance with the treatment guidelines by health-care providers as a barrier to IPT adherence. Also, none of parents/caregivers whose child contacts did not have complete adherence reported the unawareness of the benefits or length of treatment as barrier to IPT adherence in our study. The results of this study are corroborated by other studies, which indicated that provision of follow-up and service delivery were facilitators of preventive and TB treatment [36,37]. Poor follow-up and service delivery such as poor interpersonal communication between patients/caregivers and health care providers, lack of attention and support at the health facilities, difficulty for patients continue with his/her treatment if s/he missed treatment, were also found to be barriers to preventive and TB treatment adherence [38–40]. For example, studies found that when a patient missed treatment for a period and for any reason want to re-joint the TB service, s/he is jugged, insulted and sometimes requested to provide a guarantor from the community who could vouch for his/her ability and willingness to complete their course of treatment [38,41]. Therefore, to avoid those bad experiences, patients prefer to no re-join TB service. Parents/caregivers’ knowledge on the benefit of IPT and beliefs that TB is a severe and killer disease were reported as facilitators of IPT adherence. This is consistent with other studies that found that IPT completion was related to parents/caregivers’ belief about the severity of TB disease and knowledge about the benefit of IPT [16,17,42]. However, most parents/caregivers with incomplete IPT adherence in this study were knowledgeable about its benefits. The incomplete adherence observed among their child contacts could be explained by the underlying reasons for incomplete adherence. In a systematic review[36], reasons such as poverty and relocation were identified as structural factors. The latter overrides the willingness of parents/caregivers to complete IPT, despite their knowledge of the importance of adherence. Structural factors are those present in the society that influence treatment-taking behaviour, but on which the patient has little personal control.

Relocation has been identified as a barrier to IPT adherence in this study. Similar results were displayed by other studies [37,39]. Some parents/caregivers are often compelled to place their children in the care of their relatives who are wealthier than what they are. Additionally, TB patients are often displaced from their area of residence because they are either unable to pay the rents where they are staying or asked to move because they can potentially infect their neighbours. Appropriate counselling for parents/caregivers to inform health-care providers when they need to relocate and the establishment of a formal system at the health facility is needed. The communication is needed between the referral and recipient health-care providers of the child contacts to ensure they reach their destination and pursue the IPT.

In this study, poverty has been identified as a barrier to IPT. Poverty correlates with a lack of food, in fact, parents/caregivers believe that medication taken without food is harmful. Similar results have been found in other studies [43,44]. Health-care providers have to identify child contacts of poor parents/caregivers and provider them with nutritional support. Additionally, we recommend the NTP to conduct a quantitative study at the national level to access the impact of poverty on IPT adherence. Although the long duration of treatment has not been reported as a barrier to IPT, NTP has to consider the availability of shorter regimens equally effective to IPT and safer known to be associated with better adherence [10].

Incomplete adherence to IPT in this study was not associated with any individual characteristics of index cases, households or health facility characteristics as in other studies [16,17]. However, the small sample size is a limitation of this study that may have limited the ability of researchers to detect differences in the IPT uptake, with the low numbers of incomplete adherence for comparison. Nevertheless, the addition of qualitative methodology strengthened the findings by soliciting for more information, which provided an overview of barriers and facilitating factors of IPT adherence according to the views of all participants involved in IPT adherence.

Another limitation is that the data were collected by nurses. That might have compromised the qualitative data in the sense that participants might have preferred to say what the nurses wanted to hear. However, the use of nurses not involved in the treatment of child contacts or index cases, climate of trust and confidence that researchers created before starting each interview might have reduced such probability. Still, another limitation is that the research was conducted in Kigali and findings might not be generalised to elsewhere in Rwanda, especially to remote rural areas where barriers and facilitating factors to IPT adherence may be different.

Finally, the measures used to assess the IPT adherence in this study were less objective than the measures used in other studies [34,42]. These measures include pill counts or detection of INH metabolites in the urine among other things. We assumed that when a parent/caregiver attended the PHC to collect INH for his/her child, he/she also administered the medication to the child.

## Conclusions

There was a high rate of completion of IPT in this cohort of eligible child contacts living in Kigali. The success is likely attributed in part to the government's commitment through NTP to implement local interventions, especially those targeting to improve IPT adherence such as performance-based financing, free TB services and treatment, increasing numbers of PHCs and involving CHWs in the management of child contacts.

However, structural factors (poverty and relocation) were found to be the main barriers to IPT adherence. These structural factors have to be used by health-care providers to identify parents/caregivers whose child contacts are at risk of incomplete adherence, therefore, provide specific follow up adapted to their need.

## Supporting information

S1 Appendix Follow-up form for child contacts on isoniazid preventive therapy. (PDF)

S2 Appendix Interview guide English version. (PDF)

S3 Appendix Interview Kinyarwanda version. (PDF)
