# Supplementary material for: Adherence to isoniazid preventive therapy among child contacts in Rwanda: A mixed-methods study
Source: PLoS One. 2019 Feb 11;14(2):e0211934. doi: 10.1371/journal.pone.0211934 (PMC6370213; doi:10.1371/journal.pone.0211934)
Supplement: S1 Appendix — (PDF) [file pone.0211934.s001.pdf]

Follow-up form for child contacts on IPT

PS: To be filled by TB focal person during the follow up period

Child Name ---
Child Code --
Index case Name---
Index case Code--
Month of IPT collection:

Number	Question	Answers	Skip
**001**	Collection of IPT	Yes 1<br>No 2	**If 2 Q 011**
**002**	Does the child have a cough ?	Yes 1<br>No 2<br>I don't know 96	**If 2 or 96 Q 006**
**003**	How long have the child had a cough?	<1 week 1<br>1–3 weeks 2<br>3 weeks – 1 year 3<br>I don't Know 96	
**004**	Is he/she coughing up blood or blood-stained sputum?	Yes 1<br>No 2	**If 1>> Q005**
**005**	For how long has she/he coughed up blood?	<1 week 1<br>1–3 weeks 2<br>3 weeks – 1 month 3<br>> 1 month 4<br>I don't Know 96	
**006**	Has he/she had a fever?	Yes 1<br>No 2<br>I don't know 96	**If yes >> Q007**
**007**	For how long has she/he been having fever?	<1 week 1<br>1–3 weeks 2<br>>3 weeks 3<br>Don't Know 96	
**008**	Has she/he had noticeable weight loss? (≥3 kg loss in a month)	Yes 1<br>No 2<br>Don't Know 96	**If yes >> Q009**
**009**	Has she/he been sweating at night for 3 or more weeks in the last 4 weeks?	Yes 1<br>No 2<br>Don't Know 96	
**010**	Has she/he noticed any swelling and/or lumps on your neck, arm pits, or groing?	Yes 1<br>No 2<br>Don't Know 96	
**011**	Why IPT was not collected	Parents/caregivers did not come 1<br>Stock out of IPT 2<br>Others (specify) 98	

Conclusion :

☐ **Need to be transferred for X-ray and biologic exams**
☐ **No need of transfer for X-ray and biologic exams**
