# Supplementary material for: Adherence to isoniazid preventive therapy among child contacts in Rwanda: A mixed-methods study
Source: PLoS One. 2019 Feb 11;14(2):e0211934. doi: 10.1371/journal.pone.0211934 (PMC6370213; doi:10.1371/journal.pone.0211934)
Supplement: S2 Appendix — (PDF) [file pone.0211934.s002.pdf]

Study on IPT adherence among child contacts

Introduction

Good morning/good afternoon. How are you?

We are conducting a study on the evaluation of Isoniazid prophylaxis treatment and the Role of Xpert MTB/RIF test in improving the diagnosis and prevention of tuberculosis in children exposed to index Cases with Pulmonary tuberculosis in Kigali, Rwanda, for University of Rwanda, College of Medicine and Health Sciences, school of Public Health (UR-CMHS-SPH). The first part, which was quantitative, has been done (explain what was about the quantitative study even if they were part of it); and this second part is qualitative. Therefore you will be requested to give us your opinion on the provision of IPT to child contacts, your experiences with IPT (uptake, adherence, service) as parents/caregivers or healthcare providers.

Your help is highly requested and appreciated. The information you give us will be strictly confidential. You can stop the interview at any time; and if there are any questions that you do not want to answer, we can skip them. I'd like to start by asking you some general questions.

Are you ready to begin?

1. YES — CONTINUE
2. NO — STOP

The start time:---

001. your age: --
002. Your profession: --
003. Number of years doing this profession: :..
004. District: --
005. Sector: --
006: cell: --
007. Village: --
008. PHC you are using: --

Interview Guide to Parents/ Caregivers (whose children completed 6 months treatment of IPT)

1. What is your experience with bringing your child to this service? (strengths, weaknesses, challenges you faced at home, at health center, at work place [if she/he has got a job], challenges related to the child)

Probe: How did you come to know about IPT? What is your perception about IPT (strengths, weaknesses, attitude...)?

2. What is the perception of your spouse/ family about IPT (strengths, weaknesses, attitude)?
3. What motivate parents to decide that their children start and adhere to IPT ?

   Probe: You are among those whose children have completed 6 months of IPT; what motivated you?
4. There are parents whose children do not start IPT even though they are eligible; what would make it difficult for parent to start IPT?
5. There are parents whose children start the IPT, but do not complete 6 months of treatment as recommended; what makes it difficult for parents to complete 6 months treatment of IPT?
6. According to you, what can be done to improve the IPT uptake and adherence in general?

   Probe: If IPT can be given at the health center level or at the community by the community health workers; what is your preference? can you elaborate (tell us the reason of your choice)?
7. Do you have any other recommendations or comments about IPT?

****

Interview guide to parents/ Caregivers (whose children did not complete 6 months of IPT treatment)

1. What is your experience with bringing your child to this service? (strengths, weaknesses, challenges you faced at home, at health center, at work place [if she/he has got a job], challenges related to the child)

   Probe: How did you come to know about IPT? What is your perception about IPT (strengths, weaknesses, attitude...)?

2. What is the perception of your spouse/ family about IPT (strengths, weaknesses, attitude..)?
3. There are parents whose children do not start IPT even though they are eligible; what can be the reasons or barriers to the uptake?
4. There are parents whose children start the IPT, but do not complete 6 months treatment as recommended, what can be the reasons or barriers to adherence according to you?

5. According to you, what can be done to improve the IPT uptake and adherence ?
6. IPT can be given at the health center level or at the community by the community health workers; what is your preference? can you elaborate (tell us the reason of your choice)?
7. Do you have any other recommendations or comments about IPT?

****

Interview guide for Health Facilities staff

1. What is your experience in working at the IPT service?

   Probe: What is your perception about IPT (strengths, weaknesses, attitude...)?

   What challenges do you face in the IPT service ?

   What can be done to solve those challenges?
2. According to the results of our study, of 94 children who were supposed to start IPT, around 89% of them received it; what can have contributed to that result?
3. 11% of children who were supposed to start IPT did not start IPT; can you please have an idea of what contribute to that result?
4. More than 90% of children who started IPT during the period of our study completed it; according to you, what can have contributed to that result ?
5. However 10% of did not complete the IPT as recommended; can you please have idea of what contribute to that result ?
6. According to you, what can be done to improve the IPT uptake and adherence (Health facility side, client side)?
7. Do you have any other recommendations or comments about IPT?

****

FGD guide for Community Health workers

1. What is your experience in providing IPT at the community?

   Probe: How is the community IPT organized (where, when, how…)?

   What is your perception about IPT?

   What challenges do you face when giving the IPT to children?

   What are challenges faced by parents whose children must receive IPT?

2. According to the results of our study, of 94 children who were supposed to start IPT around 89% of them received it; what can have contributed to that result?
3. 11% of children, who were also supposed to start IPT did not; what can have contributed to that result?
4. More than 90% of children who started IPT during the period of our study completed it; what can have contributed to that result?
5. However 10% of them did not complete the IPT as recommended; what can have contributed to that result ?
6. According to you, what can be done to improve the IPT uptake and adherence?
7. Do you have anything else to add about IPT ?

****
