# Supplementary material for: Adherence to isoniazid preventive therapy among child contacts in Rwanda: A mixed-methods study
Source: PLoS One. 2019 Feb 11;14(2):e0211934. doi: 10.1371/journal.pone.0211934 (PMC6370213; doi:10.1371/journal.pone.0211934)
Supplement: S3 Appendix — (PDF) [file pone.0211934.s003.pdf]

Ubushakashatsi bukorerwa ku bana babana/babanye n'abarwayi b'igituntu kandi bahawe imiti ikumira igituntu ya Izoniyazide (IPT)

Mwaramutse/Mwiriwe. Amakuru yanyu?

Turi gukora ubushakashatsi bwerekeye isuzuma ry'ikoreshwa rya Izoniazide mu gukumira igituntu n'uruhare rw'ikizamini cya "Xpert MTB/RIF" mu kunoza ugusuzuma no gukumira igituntu mu bana babana/babanye n'umurwayi w'igituntu cyo mu bihaha muri Kigali mu Rwanda. Tukaba dukorera Kaminuza y'u Rwanda, Koleji y'ubuvuzi n'ubumenyi mu by'ubuzima, Ishuri ry'ubuzima rusange (UR-CMHS-SPH). Igice cya mbere cy'ubu bushakashatsi, cyakusanyije amakuru yibanda ku mibare, cyararangiye (ubasobanurire ibyerekeye ubushakashatsi bwakusanyije amakuru yibanze ku mibare n'ubwo baba barabugizemo uruhare); kandi iki gice cya kabiri kizakusanya uko abantu bumva ibintu. Iyo ni yo mpamvu turi bubasabe kuduha ibitekerezo byanyu ku byerekeye guha abana babana/babanye n'umurwayi w'igituntu umuti wa Izoniazide ngo barindwe igituntu (IPT), ibyo mwabonye kuri iyi gahunda ya IPT (ubwitabire, kubahiriza gahunda yo gufata iyi miti, ibya serivisi ziyerekeye) nk'ababyeyi/abarezi cyangwa abatanga serivisi z'ubuvuzi.

Uruhare rwanyu rurakenewe cyane kandi ni urwo kwishimirwa. Amakuru yose muduha ni ibanga rikomeye. Mushobora guhagarika ikiganiro igihe icyo ari cyo cyose; niba kandi hari ibibazo mudashaka gusubiza, dushobora kubisimbuka. Nashakaga gutangira mbabaza ibibazo rusange.

Mwa nyemerera tugatangira ikiganiro?

1. **YEGO** — **KOMEZA**
2. **OYA** — **WIKOMEZA**

Isaha ikiganiro gitangiriye : ______

001. Imyaka y'ubazwa
002. Akazi akora: --
003.Imyaka amaze akora ako kazi :..
004. Akarere
005. Umurenge
006. Akagari
007. Umudugudu
008. Ikigondera Ubuzima yivurizamo cg akoreramo

Inyoborakiganiro mu ibazwa ryimbitse ry'umubyeyi/urera umwana (ufite/urera umwana warangije amezi atandatu ateganywa muri gahunda yo kurinda abana igituntu hakoreshejwe umuti wa Izoniyazide)

1. Byagiye bikugendekera gugteigihe wazanaga umwana wawe muri iyi gahunda yo gufata imiti imurinda kwandura Igituntu?(Ndagirango umbwire aho iyi gahunda ifite imbaragacyangwa aho ifite intege nke, umbwire ingorane cg imbogamizi wahuye nazo murugo rwawe ,kw'ivuriro, mukazi[niba afite akazi],imbogamizi zerekeranye n'umwana)

   Sobanuza: Nigute wabashije kumenya ibijyanye na ghunda yo kurinda abana kwandura igituntu hakoreshejwe umuti wa Izoniyazide (IPT)? Ni iki utekereza kuri iyi gahunda yo kurinda abana kwandura igituntu hakoreshejwe umuti wa Izoniyazide (Ndagirango umbwire aho iyi gahunda ifite imbaragacyangwa aho ifite intege nke n'uko abantu bayitwaraho)?
2. Uwo mwashakanye cg umuryango wawe baba bafata bate iyi gahunda yo kurinda abana kwandura igituntuhakoreshejwe umuti wa Izoniyazide(Ndagirango umbwire aho babona imbaraga,integenke z'iyi gahunda n'uko babyitwaramo)?
3. Ni ikigishishikaza ababyeyi gufata icyemezo cyo gutangiza abanababo imiti ibarinda kwandura igituntu?

   Sobanuza: Uri umwe mu babyeyi bafite abana barangije neza amezi 6 agenwa muri gahunda yo kurinda abana kwandura igituntu hakoreshejwe umuti wa Izoniyazide;wowe ni iki cyaba cyaragushishikarije kubyubahiriza?
4. Hari ababyeyi bamwe bafite abana batinjira muri iyimuri gahunda yo kurinda abana kwandura igituntu hakoreshejwe umuti wa Izoniyazide kandi bakagombye kuyijyamo;niizihe mpamvu cg ingorane zituma bibakomerera?
5. Hari ababyeyi bamwe, abana babo batangira iyi gahunda yo kurinda abana kwandura igituntu hakoreshejwe umuti wa Izoniyazideariko ntibarangize amezi atandatu nkuko biteganywa;   ubona arink'izihe mpamvu cg ingorane zituma kurangiza amezi atandatu muri iriya gahunda bibakomerera?
6. Kubwawe,ni iki cyakorwa kugira ngo iyi gahunda yo kurinda abana kwandura igituntu hakoreshejwe umuti wa Izoniyazide yitabirwe kandi yubahirizwe inarangizwe neza n'abayitabiriye nk'uko biteganywa?

   Sobanuza: Iyi miti itangwa muri gahunda yo kurinda abana kwandura igituntu hakoreshejwe umuti wa Izoniyazideiramutseishobora gukorerwa ku kigo nderabuzima cyangwakubajyanama b' ubuzima; kubwawe nihe hakubera heza? Dudsobanurire;ni ukubera iki ariho wahitamo?
7. Hari icyo ubonatutavuze ushaka kongeraho cyerekeranye na gahunda yo kurinda abana kwandura igituntu hakoreshejwe umuti wa Izoniyazide cyangwa uburyo yarushaho kunozwa?

****

Inyoborakiganiro mu ibazwa ryimbitse ry'umubyeyi/urera umwana (ufite/urera umwana utararangije amezi atandatu ateganywa muri gahunda yo kurinda abana igituntu hakoreshejwe umuti wa Izoniyazide)

1. Byagiye bikugendekera gugte igihe wazanaga umwana wawe muri iyi gahunda yo gufata imiti imurinda kwandura Igituntu? (Ndagirango umbwire aho iyi gahunda ifite imbaragacyangwa aho ifite intege nke, umbwire ingorane cg imbogamizi wahuye nazo mu rugo rwawe , kw'ivuriro, mu kazi [niba afite akazi], imbogamizi zerekeranye n'umwana)

Sobanuza: Ni gute wabashije kumenya ibijyanye na ghunda yo kurinda abana kwandura igituntu hakoreshejwe umuti wa Izoniyazide (IPT)? Ni iki utekereza kuri iyi gahunda ya yo kurinda abana kwandura igituntu hakoreshejwe umuti wa Izoniyazide (Ndagirango umbwire aho iyi gahunda ifite imbaragacyangwa aho ifite intege nken'uko abantu bayitwaraho)?

2. Uwo mwashakanye cg umuryango wawe baba bafata bate iyi gahunda yo kurinda abana kwandura igituntu hakoreshejwe umuti wa Izoniyazide (Ndagirango umbwire aho iyi gahunda ifite imbaragacyangwa aho ifite intege nken'uko babyitwaramo)?

3. Hari ababyeyi bamwe bafite abana batinjira muri iyimuri gahunda yo kurinda abana kwandura igituntu hakoreshejwe umuti wa Izoniyazide kandi bakagombye kuyijyamo; ni izihe mpamvu cg ingorane zituma batazana abana babo gufata iyi miti?
4. Hari ababyeyi bamwe, abana babo batangira iyi gahunda yo kurinda abana kwandura igituntu hakoreshejwe umuti wa Izoniyazideariko ntibarangize amezi atandatu nk'uko biteganywa; ubona ari nk'izihe mpamvu cyangwa ingorane zitumaabana babobatarangiza iyo miti nk'uko byategsnijwe?
5. Kubwawe, ni iki cyakorwa kugira ngo iyi gahunda yo kurinda abana kwandura igituntu hakoreshejwe umuti wa Izoniyazide yitabirwe kandi yubahirizweinarangizwe neza n'abayitabiriye nk'uko biteganywa?
6. Iyi miti itangwa muri gahunda yo kurinda abana kwandura igituntu hakoreshejwe umuti wa Izoniyazide, ishobora gukorerwa ku kigo nderabuzima cyangwa ku bajyanama b' ubuzima; kubwawe ni he hakubera heza? Dudsobanurire; ni ukubera iki ariho wahitamo ?
7. Hari icyoubona tutavuze ushaka kongeraho cyerekeranye na gahunda yo kurinda abana kwandura igituntu hakoreshejwe umuti wa Izoniyazidecyangwa uburyo yarushaho kunozwa?

****

Inyoborakiganiro mu ibazwa ryimbitse ry'umukozi wo ku ivuriro

1. Ni ayahe makuru wadusangiza y'uko bimeze/umerewe mu kazi ukorera muri iyi serivisiyo kurinda abana kwandura igituntu hakoreshejwe umuti wa Izoniyazide?

Sobanuza: Gahunda yo kurinda abana kwandura igituntu hakoreshejwe umuti wa Izoniyazide, uyibona ute/uyitekerezaho iki (tubwire aho iyi gahunda ifite imbaragacyangwa aho ifite intege nke, uko ababagana cyanwa mwe muyitwaraho)?

Ni izihe mbogamizi ugira muri iyi serivisi yo kurinda abana kwandura igituntu hakoreshejwe umuti wa Izoniyazide?

Ni iki cyakorwa kugirango izombogamizi zikurweho?

2. Ubushakashatsi twakoze bwerekanye ko mu bana 94 bakagombye kuba baratangiye imiti yaIzoniyazide yo kubarinda kwandura igituntu, 89% bayibonye. Ni iki cyaba cyaratumye ibyo bibasha kugerwaho?

3. Mu bana bari bateganyijwe gutangira imiti ya Izoniyazide yo kubarinda kwandura igituntu,11% ntibayitangiye; byaba byaratewe n'iki?
4. Mugihe ubu bushakashatsi bwacubwamaze bukorwa, mubana batangiye imiti ya Izoniyazide yo kubarinda kwandura igituntu,abarenga90% bayirangije neza.Ni iki cyaba cyaratumye ibyo bibasha kugerwaho?
5. N'ubwo bimezebityo,mubanabatangiye imiti ya Izoniyazide yo kubarinda kwandura igituntu,10% ntibayirangije nk'uko biteganywa;byaba byaratewe n'iki?
6. Kubwawe, ni iki cyakorwa kugira ngo iyi gahunda yo kurinda abana kwandura igituntu hakoreshejwe umuti wa Izoniyazide yitabirwe kandi yubahirizwe inarangizwe neza n'abayitabiriye nk'uko biteganywa (icyakorwa ku ruhande rw'ivuriro no ku ruhande rw'abarigana)?
7. Hari icyo ubona tutavuze ushaka kongeraho cyerekeranye na gahunda yo kurinda abana kwandura igituntu hakoreshejwe umuti wa Izoniyazide cyangwa uburyo yarushaho kunozwa?

****

Inyoborakiganiro mu ibazwa ry'itsinda ry'abajyanama b'ubuzima

1. Ni ayahe makuru wadusangiza y'uko bimeze/umerewe mu kazi ukorera mu mudugudu ko kurinda abana kwandura igituntu hakoreshejwe umuti wa Izoniyazide?
   - Ni gute mu mudugudu mutanga imiti irinda abanakwandura igituntu (Nihe muyitangira ?Niryari muyitanga?Nigute muyitanga?)
   - Gahunda yo kurinda abana kwandura igituntu hakoreshejwe umuti wa Izoniyazide, uyibona ute/uyitekerezaho iki?
   - Ni izihe mbogamizi muhura na zo mugutanga imiti irinda abana kwandura igituntu?
   - Ni izihe mbogamiziababyeyibahura nazo mugihe abana babo bagomba gufata imiti ibarinda kwandura igituntu?
2. Ubushakashatsi twakoze bwerekanye komu bana 94 bakagombye kuba baratangiyeimiti ya Izoniyazide yo kubarinda kwandura igituntu, 89% bayibonye. Niiki cyaba cyaratumye ibyo bibasha kugerwaho?

3. Mu bana bari bateganyijwe gutangira imiti ya Izoniyazide yo kubarinda kwandura igituntu,11% ntibayitangiye;byaba byaratewe n'iki?

4. Mugihe ubu bushakashatsi bwacubwamaze bukorwa, mubana batangiye imiti ya Izoniyazide yo kubarinda kwandura igituntu, abarenga 90% bayirangije neza.Ni iki cyaba cyaratumye ibyo bibasha kugerwaho?
5. N'ubwo bimezebityo, mubanabatangiye imiti ya Izoniyazide yo kubarinda kwandura igituntu,10% ntibayirangije nk'uko biteganywa; byaba byaratewe n'iki?
6. Kubwanyu, ni iki cyakorwa kugira ngo iyi gahunda yo kurinda abana kwandura igituntu hakoreshejwe umuti wa Izoniyazide yitabirwe kandi yubahirizwe inarangizwe neza n'abayitabiriye nk'uko biteganywa?
7. Kubijyanye na gahunda yo kurinda abana kwandura igituntu hakoreshejwe umuti wa Izoniyazide, hari icyoubona tutavuze ushaka kongeraho ?

Murakoze kwitabira gahunda yacyu.
